# High water content in primitive continental flood basalts

**DOI:** 10.1038/srep25416

**Published:** 2016-05-04

**Authors:** Qun-Ke Xia, Yao Bi, Pei Li, Wei Tian, Xun Wei, Han-Lin Chen

**Affiliations:** 1 School of Earth Sciences, Zhejiang University, Hangzhou, China; 2 School of Earth and Space Sciences, University of Science and Technology of China, Hefei, China; 3 School of Earth and Space Sciences, Peking University, Beijing, China; 4 Key Laboratory of Marine Geology and Environment, Institute of Oceanology, Chinese Academy of Sciences, Qingdao, China

## Abstract

As the main constituent of large igneous provinces, the generation of continental flood basalts (CFB) that are characterized by huge eruption volume (>10^5^ km^3^) within short time span (<1–3 Ma) is in principle caused by an abnormally high temperature, extended decompression, a certain amount of mafic source rocks (e.g., pyroxenite), or an elevated H_2_O content in the mantle source. These four factors are not mutually exclusive. There are growing evidences for high temperature, decompression and mafic source rocks, albeit with hot debate. However, there is currently no convincing evidence of high water content in the source of CFB. We retrieved the initial H_2_O content of the primitive CFB in the early Permian Tarim large igneous province (NW China), using the H_2_O content of ten early-formed clinopyroxene (cpx) crystals that recorded the composition of the primitive Tarim basaltic melts and the partition coefficient of H_2_O between cpx and basaltic melt. The arc-like H_2_O content (4.82 ± 1.00 wt.%) provides the first clear evidence that H_2_O plays an important role in the generation of CFB.

As the main constituent of continental large igneous provinces (LIPs)^1^, continental flood basalts (CFB) are characterized by huge eruptive volumes within a relatively short time span. The estimated eruptive basalt volumes range from ~2 × 10^5 ^ km^3^ for the Columbia River Basalts to > ~ 2 × 10^6 ^ km^3^ for the Siberian Traps^2^,^3^. The time span is usually as short as <1–3 My^4^,^5^,^6^,^7^,^8^. These features imply a special geodynamic process in the mantle and may trigger prominent environmental effects (climate change, mass extinction, etc.) and contribute to the formation of giant metal ore deposits^9^.

In principle, the generation of CFB requires an abnormally high temperature, extended decompression, a certain amount of mafic rocks in the mantle source, or the addition of H_2_O and/or CO_2_ into the mantle source^10^,^11^. These four factors are not mutually exclusive, and it is likely that several or all factors contribute together to generate CFB. The elevated H_2_O and/or CO_2_ content allows melting to start in the deeper mantle and enlarges the whole melting regime, consequently contributing to the enormous melt. The CO_2_ content is much less than the H_2_O content in the mantle^12^, and the magnitude of the lowering solidus of the upper mantle by CO_2_ is less than that of H_2_O^13^. Therefore, adding H_2_O is expected to be more important for the genesis of CFB. High temperature, decreased pressure and mafic source lithology have been extensively discussed, albeit debated, for three decades^14^,^15^,^16^,^17^,^18^,^19^, but the evidence of high H_2_O content is scarce.

Indeed, there were attempts to obtain H_2_O content of mineral-hosted melt inclusions in CFB, but the extent to which they can reflect the initial H_2_O content of primitive basaltic magmas (i.e. the magmas that after being extracted from their source regions have experienced little modification) was controversial. Stefano *et al.*^20^ and Cabato *et al.*^21^ measured melt inclusions hosted by olivine phenocrysts in the CFB of the Yellowstone hotspot track and the Columbia River, respectively. They found that the H_2_O content in melt inclusions is as high as 2.4 wt.% for the Yellowstone and 3.3 wt.% for the Columbia River. However, the melt inclusions with the highest H_2_O content are not hosted by the earliest-formed (i.e., with highest Fo value) olivine phenocrysts, so they may represent the H_2_O content of the evolved melts rather than that of the initial ones. Ten melt inclusions in olivine phenocrysts from the Siberian Traps basalts have H_2_O contents ranging from 0.01 wt.% to 1.6 wt.%^22^,^23^, almost falling in the range of mid-ocean ridge basalts (MORB, ~0.1–0.3 wt.%)^24^,^25^,^26^,^27^,^28^,^29^ and ocean island basalts (OIB, 0.3–1.0 wt.%)^29^,^30^,^31^,^32^,^33^. However, the possibility of loss of H_2_O due to late-stage degassing processes was not evaluated for the Siberian melt inclusions.

In addition, Michael *et al.*^34^ and Wallace *et al.*^35^ analysed basaltic glasses of the Ontong Java and Kerguelen oceanic plateaus (the oceanic counterpart of CFB), respectively. The H_2_O content in these glasses ranges from 0.13 to 0.49 wt.% for the Ontong Java and from 0.24 to 0.69 wt.% for the Kerguelen oceanic plateaus and is only slightly higher than that of MORB. However, the low Mg# (=100 Mg/(Mg + Fe) mol.) of 40–60 indicated that the basaltic glasses they analysed are evolved melts, again arguing against the representativeness of the initial melts. Overall, there is currently no unarguable evidence to show whether the generation of CFB is related to the high H_2_O content of the mantle source.

Here, we calculate the initial H_2_O content of the early Permian Tarim CFB in NW China (>200,000 km^2^ flood basalts)^9^,^36^ using the H_2_O content of clinopyroxene (cpx) macrocrysts crystallized from the primitive Tarim flood basalts and the H_2_O partition coefficient between cpx and basaltic melt. The inferred high H_2_O content in the initial basaltic melt provides the first firm evidence that H_2_O plays an important role in the generation of CFB.

## Samples and previous study

**Figure 1:**
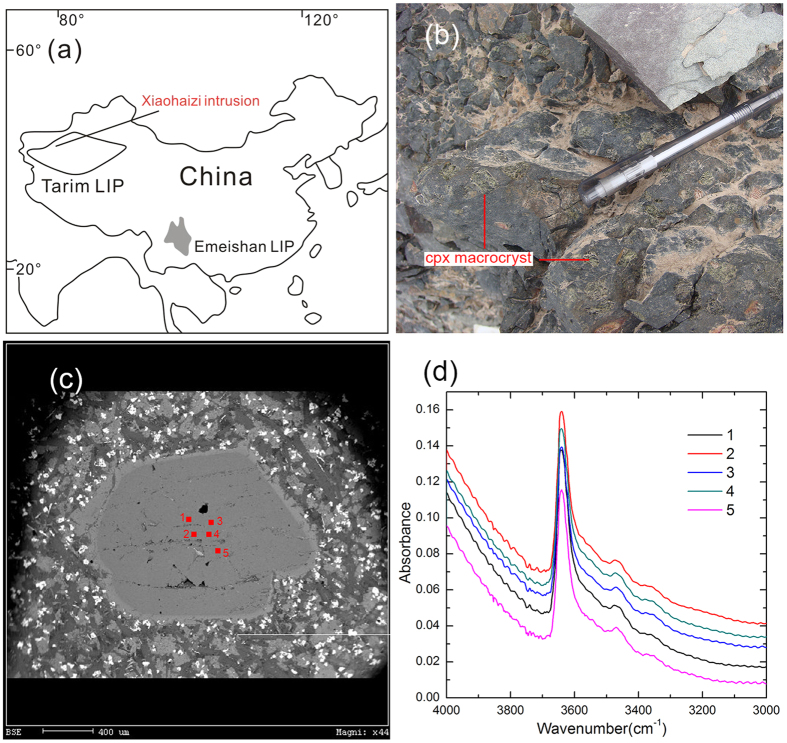
(**a**) Location of the Tarim LIP and Xiaohaizi intrusion. This figure is created by the software CorelDRAW(R) Graphics Suite 12 (http://www.corel.com/cn/). (**b**) Clinopyroxene macrocrysts in the Xiaohaizi dyke. (**c**) Backscattered electron (BSE) image of a typical clinopyroxene macrocryst (XU06-09) which has a gray core and a thin and bright rim. Red squares are five spots for FTIR and EPMA analyses. (**d**) IR spectra of five spots in c showing similarities both in OH band positions and heights.

Many cpx macrocrysts (1–15 mm of grain size) were hosted by one basaltic dyke that crosscuts to the Early Permian (~280 Ma) Xiaohaizi wehrlite intrusion in the Tarim large igneous province, NW China (Fig. 1a,b). They are fresh and usually prismatic and sub- to euhedral shapes (Fig. 1c), and they commonly have a high-Mg (Mg# = 80–89) core and a thin low-Mg rim (Mg# down to 70) that is resulted from the interaction with the host basalt^37^. Wei *et al.*^37^ carried out a detailed geochemical analysis on these macrocrysts. These cpx generally have low TiO_2_ (0.26–1.09 wt.%), Al_2_O_3_ (1.15–3.10 wt.%) and Na_2_O (0.16–0.37 wt.%) compared to the cpx in mantle peridotites (0.31–2.50 wt.% TiO_2_, 1.32–12.55 wt.% Al_2_O_3_ and 0.2–1.90 wt.% Na_2_O), so they are not likely to be xenocrysts from mantle peridotites. The macrocrysts have strong resorption textures and are not in chemical equilibrium with the host basaltic dyke, arguing against a phenocryst genesis. In addition, the cpx macrocrysts define a coherent compositional trend (e.g., negative correlations between Mg# and Ti, Al, Na, La, Nd, Yb)^37^ with the cpx from the wehrlites crosscut by the basaltic dyke hosting the cpx macrocrysts, and these cpx have identical trace element distribution patterns, demonstrating a comagmatic origin. Accordingly, these macrocrysts have been ascribed to be antecrysts that crystallized from the earlier and more primitive melts and have been reincorporated into the host basalt dyke before intrusion. High-Mg values indicate that the cpx macrocrysts were formed from a nearly primary basaltic melt. Although an assimilation and fractional crystallization process may operate during the formation of the Xiaohaizi intrusion that was evidenced by higher ^87^Sr/^86^Sr_i_ (0.7038–0.7041) and lower εNd_i_ (1.0–1.9), the preservation of the high-Mg feature and depleted Sr-Nd isotope compositions (^87^Sr/^86^Sr_i_ = 0.7035–0.7037, and εNd_i_ = 4.5–4.8) suggests that the cores of these cpx macrocrysts may have recorded the composition of the primitive Tarim basaltic melts, with little crustal contamination^37^. The Cpx macrocrysts in this paper were from the same dyke studied by Wei *et al.*^37^.

## Results

The chemical composition and H_2_O content in 10 cpx grains were obtained by an electron probe micro-analyzer (EPMA) and a Fourier transform infrared spectrometer (FTIR), respectively (see Methods). Wei *et al.*^37^ have shown that the rims of the Tarim cpx macrocrysts may have reacted with the host basalt, so only the clean core area of each cpx grain was measured here, in order to retrieve the information about the initial and primitive basaltic melts. 4–6 clean analysed spots in the core area were selected to run EPMA and FTIR for each grain (Fig. 1c), and in individual grains they show same chemical compositions and IR spectra (Fig. 1d). The average values of the analysed spots of each grain were, therefore, used to represent the element and H_2_O contents of that grain. Ten cpx grains have TiO_2_ (0.39–0.66 wt.%), Al_2_O_3_ (1.23–1.77 wt.%) and Na_2_O (0.15–0.26 wt.%) (Table 1), which is within the range reported by Wei^37^. The cpx Mg# values are 85.2 to 87.8 (Table 1), corresponding to a Mg# of ~70 for the equilibrated basaltic melts using the experimental Mg-Fe partition coefficient (0.34 ± 0.04)^38^. This suggests that the analysed cpx grains were crystallized from a nearly primary basaltic source^39^, in agreement with the trace element and Sr-Nd isotope characteristics of the Tarim cpx macrocrysts^37^.

**Figure 2:**
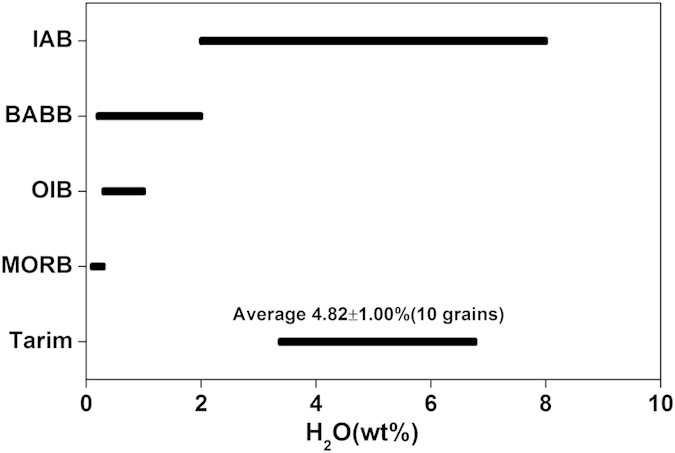
Comparison of the initial H_2_O contents of the Tarim primitive basalts with those of MORB, OIB, BABB and IAB. The range of MORB, OIB, BABB and IAB is from references 24, 25, 26, 27, 28, 29, 29, 30, 31, 32, 33, 43, 44, 45 and 46, 47, 48, respectively.

The IR absorption spectra of the Tarim cpx can be subdivided into four groups, namely: 3630–3620 cm^−1^, 3540–3520 cm^−1^, 3470–3450 cm^−1^ and 3360–3350 cm^−1^ (Fig. 1d). The band at 3630 ~ 3620 cm^−1^ and 3540–3520 cm^−1^ is always strongest and weakest, respectively, and the band at 3360–3350 cm^−1^ occurs in few grains, consistent with the structural OH bands in the cpx phenocrysts in Mesozoic-Cenozoic basalts of eastern China^40^,^41^,^42^. The calculated H_2_O contents of 10 cpx grains are 300–550 wt. ppm (Average: 384 ± 83 wt. ppm), and the calculated H_2_O contents of the equilibrated basaltic melts are 3.69 wt.% to 6.61 wt.% (Average: 4.82 ± 1.00 wt.%) (Table 1). Within the 40% uncertainty (see Methods), the H_2_O contents of the equilibrated melts do not show significant variations when the Mg# of the cpx varies from 85.2 to 87.8, suggesting that the H_2_O content in the magma system remained almost constant at the early stage of magma evolution. Therefore, it is reasonable to use the calculated H_2_O content of the melts equilibrated with the analysed cpx to represent the H_2_O content of the initial and primitive Tarim basaltic melt. Although bearing an uncertainty of up to 40%, such an H_2_O content is apparently higher than those of MORB, OIB and back-arc basin basalts (BABB, 0.2–2.0 wt.%)^43^,^44^,^45^ and falls in the range of island arc basalts (IAB, 2.0–8.0 wt.%)^46^,^47^,^48^ (Fig. 2).

## Discussion

The arc-like H_2_O contents in the early Permian Tarim primary basaltic melts indicate an addition of water from subduction-related processes. In the mid-Proterozoic, the Tarim was surrounded by subduction zones^49^. In addition, ophiolite mélanges and arc-like magmatic events along the northern margin of the Tarim were dated at 600–418 Ma and 422–363 Ma, respectively, suggesting an active convergent margin^50^. These subduction processes may have provided water to the source of the Tarim Early-Permian basalts. However, the Tarim basalts do not display arc-like geochemical signatures (i.e., LILEs-enrichment and HFSEs-depletion)^36^,^51^,^52^,^53^,^54^. This suggests that the extra water in the source of the Tarim basalts was not from the released fluids from the subducting plates, but was instead from the dehydrated plates stagnated in the deep earth. Experimental and natural investigations have demonstrated that minerals (cpx, garnet, olivine, etc.) in dehydrated plates can carry at least several thousands ppm (wt.) of H_2_O into the Earth’s mantle^55^,^56^. Garnets and omphacites from ultra-high pressure matamorphic eclogites have also been shown containing ~2000–3000 ppm wt. H_2_O^57^,^58^. If we consider that (1) the partition coefficient of H_2_O between the mantle rock (peridotite, eclogite, pyroxenite) and melt is ~0.01^59^ and (2) the degree of partial melting of the Tarim basalts is <10%^51^,^53^, then <5000 ppm wt. H_2_O in the source can produce 5% H_2_O in basaltic melts, regardless of the melting model (batch or fractional) involved.

The upper mantle can accommodate several hundred ppm (wt.) of H_2_O^56^,^60^, and the lower mantle contains much less^61^. Only the mantle transition zone (MTZ) can contain up to >1 wt.% H_2_O^55^,^62^. Several thousands ppm (wt.) of H_2_O in the source of the Tarim basalts is, therefore, likely from the MTZ where the subducted plates stagnated and provided water^63^. If so, the classic core-mantle boundary-derived plume model^15^,^64^ cannot be applied to the Tarim large igneous province.

In conclusion, the high water content in the primary early Permian Tarim basalts provides clear evidence that water, in addition to the temperature, pressure and source lithology, plays an important role in the generation of continental flood basalts. Furthermore, when high water content is considered, abnormally high temperature and extended decompression that are two critical factors in the widely accepted mantle plume model^65^ are not always to be prerequisites in the generation of CFB (and LIPs).

## Methods

The H_2_O content of cpx was determined with a Nicolet iso50 FTIR coupled with a Continuμm microscope in School of Earth Sciences, Zhejiang University, following the unpolarized method described in Xia *et al.*^40^. For each cpx grain, several analysed spots (~50 μm × 50 μm) were set in the clean core area and they display almost same spectra, therefore the average spectrum was used to calculate the H_2_O content of that grain. The modified Beer-Lambert law [*c* = *A*/(*I* × *t*)] was used to calculate to H_2_O content, in which *c* is the content of water (H_2_O ppm wt.), *A* is the total integral absorption of OH bands (cm^−2^) that is 3 times of the integral area of unpolarized absorption^66^, *I* is the integral specific absorption coefficient (7.09 ppm^−1 ^cm^−2^)^67^, *t* is the thickness (cm). The uncertainty of H_2_O content is less than 30%^40^.

The major element contents of cpx were analysed using a Shimadzu EPMA 1600 at University of Science and Technology of China. The 15 kV accelerating voltage, 20 nA beam current and 1 μm beam diameter were used. Standards are natural minerals and synthetic oxides. Data correction was obtained by a program based on the ZAF procedure. The reproducibility is <1% for elements with concentration >5% and <3% for elements with concentration > 1%. The analysed points were set within the FTIR analysed area. Several points in each cpx grain have homogeneous element contents, and the average values were used (Table 1).

The H_2_O content of the basaltic melts equilibrated with cpx is estimated by the H_2_O content of cpx and the H_2_O partition coefficients (Dcpx/melt) between cpx and melt. Dcpx/melt can be calculated by the equation 10 in O’Leary *et al.*^68^: D = exp(−4.2 + 6.5^*^X(^iv^Al)-X(Ca)), where X(^iv^Al) and X(Ca) are the concentration of octahedrally coordinated Al^3+^ in tetrahedral site and Ca^2+^ in cpx calculated on the basis of 6 oxygen. This equation was derived by compiling experimental results run at temperatures between 1025 °C and 1440 °C, pressures between 0.5–5.0 GPa, melt H_2_O contents between 1.09 wt.% and 24.9 wt.%, and cpx ^iv^Al between 0.002 and 0.306. Considering the uncertainties from Dcpx/melt (~10%)^68^ and H_2_O content in cpx (<30%), the total uncertainty of H_2_O contents in melts is estimated to be less than 40%^40^,^41^,^42^.

## Additional Information

**How to cite this article**: Xia, Q.-K. *et al.* High water content in primitive continental flood basalts. *Sci. Rep.* **6**, 25416; doi: 10.1038/srep25416 (2016).

## Figures and Tables

**Table 1:** Chemical composition and H_2_O content of the Tarim clinopyroxenes and H_2_O content of the corresponding basaltic melts.

Sample	xu05-01	xu05-02	xu05-03	xu06-01	xu06-02	xu06-03	xu06-04	xu06-07	xu06-09	xu06-10	Average	1 SD
wt.%												
SiO_2_	53.76	53.43	53.07	53.81	53.94	53.63	53.79	53.87	54.16	53.58		
TiO_2_	0.41	0.39	0.58	0.43	0.55	0.52	0.47	0.59	0.48	0.66		
Al_2_O_3_	1.33	1.49	1.70	1.23	1.43	1.53	1.51	1.59	1.55	1.77		
Cr_2_O_3_	0.36	0.44	0.32	0.52	0.24	0.68	0.26	0.33	0.33	0.24		
FeO	4.88	4.41	5.19	4.31	4.75	4.32	4.54	4.95	4.66	5.12		
NiO	0.018	0.08	0.03	0.011	0.005	0.061	0.031	0.037	0.029	0.065		
MnO	0.079	0.08	0.09	0.082	0.068	0.048	0.067	0.084	0.079	0.062		
MgO	16.98	17.43	16.69	17.44	17.10	17.03	16.54	16.78	17.05	16.64		
CaO	21.84	21.51	21.98	21.79	21.91	21.78	21.63	21.71	21.98	21.89		
Na_2_O	0.15	0.18	0.19	0.20	0.21	0.26	0.23	0.19	0.19	0.22		
K_2_O	0.00	0.00	0.01	0.00	0.00	0.01	0.00	0.00	0.00	0.01		
Total	99.79	99.44	99.84	99.83	100.20	99.86	99.07	100.13	100.49	100.26		
Mg#	86.1	87.6	85.2	87.8	86.5	87.5	86.7	85.8	86.7	85.3		
^iv^Al	0.029	0.037	0.048	0.032	0.032	0.037	0.019	0.032	0.031	0.041		
Ca	0.858	0.847	0.866	0.854	0.856	0.854	0.853	0.850	0.856	0.857		
D(cpx/melt)	0.0077	0.0082	0.0086	0.0079	0.0079	0.0081	0.0072	0.0079	0.0078	0.0083		
Cpx H_2_O (ppm)	380	310	380	385	300	300	350	500	380	550	384	83
melt H_2_O(wt.%)	4.94	3.78	4.40	4.89	3.82	3.69	4.84	6.32	4.89	6.61	4.82	1.00

Mg# = 100Mg/(Mg + Fe), ^iv^Al and Ca are atomic numbers calculated based on 6 oxygen atoms. D(cpx/melt) is calculated by the equation 10 in O’Leary *et al.*^68^, Cpx H_2_O is measured by FTIR, melt H_2_O = Cpx H_2_O/D(cpx/melt).
